#  

**DOI:** 10.1111/jcmm.16354

**Published:** 2021-02-23

Retraction: MircoRNA‐1275 promotes proliferation, invasion and migration of glioma cells via SERPINE1. Dong‐Mei Wu, Shan Wang, Xin Wen, Xin‐Rui Han, Yong‐Jian Wang, Shao‐Hua Fan, Zi‐Feng Zhang, Qun Shan, Jun Lu, Yuan‐Lin Zheng. 2018; 22: 4963–4974. https://doi.org/10.1111/jcmm.13760

The above article from Journal of Cellular and Molecular Medicine, published online on 19 July 2018 on Wiley Online Library (wileyonlinelibrary.com) in Volume 22, pp. 4963‐4974, has been retracted by agreement between the authors, the journal Editor‐in‐Chief and John Wiley & Sons Ltd. The retraction has been agreed following an investigation based on allegations raised by a third party. The authors asked to retract this manuscript as raw experimental data were not accessible anymore and thus the validity of the overall results could not be confirmed.

